# Supplementary material for: Identification of photoperiod-induced specific miRNAs in the adrenal glands of Sunite sheep (Ovis aries)
Source: Front Vet Sci. 2022 Jul 22;9:888207. doi: 10.3389/fvets.2022.888207 (PMC9354845; doi:10.3389/fvets.2022.888207)
Supplement: Supplementary file 1 [file Data_Sheet_1.ZIP › Supplementary file/miRDeep2 file.zip!/miRDeep2 file/Know_Structure/SPLP42A3/oar-mir-26b.pdf]

miRBase precursor	: oar-mir-26b
Total read count	: 152215
oar-miR-26b read count	: 152161
remaining reads	: 54

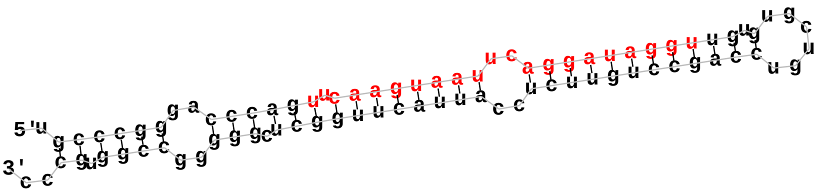

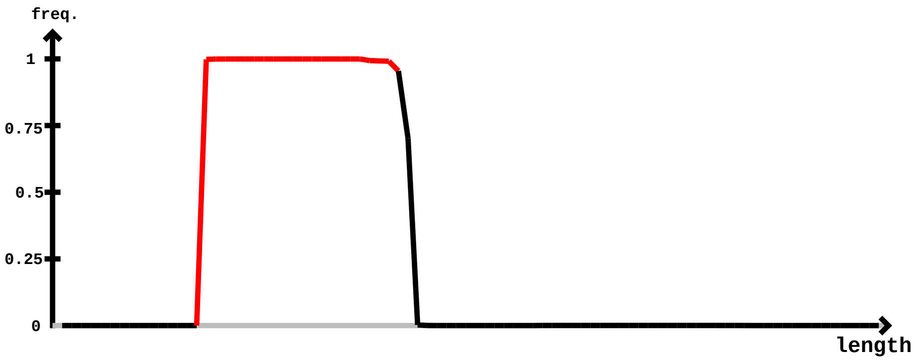

oar-miR-26b

5'- ugcccgggacccaguucaaguaauucaggauaggcuugugugcuguccagccuguucuccauuacuuggcucgggggccggugccc -3'	exp		
.((((((..((((((.((((((((..(((((((((((.(......))))))))))))..))))))))))).))).)))).))..	reads	mm	sample
.............guucaaguaauucaggauaggu..	1	0	seq
..............uucaaguaauucagUau..	1	1	seq
..............uucaaguaauucaggau..	716	0	seq
..............uucaaguaCuucaggau..	1	1	seq
..............uucaaguaaGucaggau..	1	1	seq
..............Aucaaguaauucaggau..	4	1	seq
..............uCcaaguaauucaggau..	3	1	seq
..............uucaagAaauucaggau..	2	1	seq
..............uucaaguaaCucaggau..	2	1	seq
..............uucaGguaauucaggau..	1	1	seq
..............uucaUguaauucaggau..	4	1	seq
..............uucaagGaauucaggau..	1	1	seq
..............uucaaguaauucaggaC..	85	1	seq
..............uucCaguaauucaggau..	60	1	seq
..............uucaaguaauucaggUu..	2	1	seq
..............uucaaguaauuUaggau..	1	1	seq
..............uucaaguGauucaggau..	1	1	seq
..............uuUaaguaauucaggau..	4	1	seq
..............uucaaguaauucagAau..	1	1	seq
..............uucaaguaauucaggaA..	13	1	seq
..............uucaagCaauucaggau..	1	1	seq
..............uucCaguaauucaggaua...	7	1	seq
..............uucaaguaauucaggaua...	212	0	seq
..............uucaaguaauucaggaAa...	1	1	seq
..............uucaaguaauucagAaua...	1	1	seq
..............uucaaguaauucaggauag..	138	0	seq
..............uucaaguaauucaggauagg...	5562	0	seq
..............uucaaguaauucaggauaggu..	38416	0	seq
..............uucaaguaauucaggauagguu...	106331	0	seq
..............uucaaguaauucaggauagguug..	317	0	seq
..............uucaaguaauucaggauagguugu...	41	0	seq
...............ucaaguaauucaggaua...	1	0	seq
...............ucaaguaauucaggauagg...	5	0	seq
...............ucaaguaauucaggauaggu..	37	0	seq
...............ucaaguaauucaggauagguu...	144	0	seq

oar-miR-26b

```
ugcccgggacccaguucaaguaauucaggauagguugugugcuguccagccuguucuccauuacuuggcucgggggccggugccc
...............ucaaguaauucaggauagguug................................................	7	0	seq
...............ucaaguaauucaggauagguugu...............................................	1	0	seq
................caaguaauucaggauagg...................................................	1	0	seq
................caaguaauucaggauaggu..................................................	4	0	seq
................caaguaauucaggauagguu.................................................	19	0	seq
................caaguaauucaggauagguug................................................	1	0	seq
.................aaguaauucaggauagg...................................................	1	0	seq
.................aaguaauucaggauagguu.................................................	9	0	seq
....................................gugugcuguccagccuguucuccauuacuuggcuc..............	2	0	seq
.................................................ccuguucuccauuacuugg.................	3	0	seq
.................................................ccuguucuccauuacuuggc................	2	0	seq
.................................................ccuguucuccauuacuuggcu...............	16	0	seq
.................................................ccuguucuccauuacuuggcuc..............	28	0	seq
.................................................ccuguucuccauuacuuggcucg.............	1	0	seq
..................................................cuguucuccauuacuuggcuc..............	2	0	seq
```
